# Application Prospects of Alginate Oligosaccharides in Regulating Maize Stress Tolerance: A Review

**DOI:** 10.3390/ijms27198663

**Published:** 2026-09-28

**Authors:** Qifeng Wu, Lizhi Wang, Kai Liu, Limin Yu, Ruxiao Bai

**Affiliations:** 1 Institute of Field Water Conservancy, Soil and Fertilizer Research, Xinjiang Academy of Agricultural and Reclamation Sciences, Shihezi 832000, China; 18615177887@163.com (L.Y.); brx_shz@163.com (R.B.); 2 Modern Protected Horticulture Technology Innovation Center of Xinjiang Production and Construction Corps, Shihezi 832000, China; 3 Faculty of Hydraulic Engineering, Environment and Oceanography, Ludong University, Yantai 264025, China; yantailizhi@126.com; 4 Dongying Research Institute for Oceanography Development, Dongying 257091, China; 5 School of Information Science and Engineering, Shandong Agriculture and Engineering University, Jinan 250100, China

**Keywords:** alginate oligosaccharides, maize, stress tolerance, regulate mechanism, production

## Abstract

Maize (*Zea mays* L.) plays an irreplaceable role in food, feed, and energy security. However, abiotic stresses, including drought, high temperature, salinity, and waterlogging, driven by climate warming, cause yield losses of 20–30% of global maize production every year, posing a serious threat to global food security and human development. Traditional approaches such as genetic improvement, agronomic optimization, and chemical regulation struggle to address the multiple stresses affecting maize. Alginate oligosaccharides (AOSs)—linear oligosaccharides with degrees of polymerization of 2–20—can systematically activate plant immunity, reshape root architecture, enhance antioxidant defense, and regulate ion homeostasis to simultaneously achieve yield enhancement and stress tolerance. This review assesses global maize production and abiotic stress-induced losses and analyzes the limitations of existing yield stabilization technologies. The molecular physiological networks through which AOSs regulate maize yield formation and stress adaptation are also elucidated. Furthermore, through bibliometric analysis, we reveal the evolutionary trajectory from phenotypic observation to mechanistic dissection and identify current knowledge gaps. Finally, we propose future research priorities, including AOS receptor identification, model applications of AOSs, and combined-stress regulation by AOSs. This review provides theoretical foundations and practical pathways for incorporating AOSs into sustainable maize development.

## 1. Introduction

Maize (*Zea mays* L.) is the world’s most widely produced cereal crop. According to FAO statistics, over 1.2 billion people in sub-Saharan Africa, Latin America, and South Asia rely on maize as a staple food [1,2,3]. Additionally, approximately 60% of maize enters the feed processing chain, indirectly supporting the global supply of animal protein [4]. These figures highlight the central role of maize in global food, feed, and energy security. However, over the past two decades, the average annual growth rate of global maize yield has declined from approximately 2.5% during the “Green Revolution” era to less than 0.8% [5,6]. Meanwhile, abiotic stresses such as drought, high temperature, salinization, and waterlogging, driven by climate change, are becoming more frequent and complex. These stresses cause annual global maize yield losses of 250–350 million tons, representing approximately 20–30% of total production [7,8,9]. Conventional mitigation strategies face clear limitations: genetic improvement is slowed by the polygenic inheritance of stress tolerance; high-input agronomic measures are difficult to implement in developing regions; and chemical plant growth regulators have narrow application windows with potential negative effects [10,11,12]. Developing novel inputs that combine signal-regulatory functions, low cost, broad adaptability, and environmental compatibility may offer a viable pathway to address these challenges.

Alginate oligosaccharides (AOSs) are linear oligosaccharides with degrees of polymerization of 2–20, generated via enzymatic degradation of alginate. AOSs can affect crop growth at ppm concentrations. As signaling molecules, AOSs perceive and transduce signals via plant plasma membrane receptors, systematically activating immunity, optimizing root architecture, enhancing antioxidant defense, and regulating ion homeostasis [13,14,15]. Moreover, even at high doses, AOSs do not negatively affect maize (corn) growth. These properties distinguish AOSs from traditional plant growth regulators. However, systematic research on AOSs in field crops such as maize started relatively recently, with a particular lack of in-depth mechanistic analyses and precision application systems adapted to stress conditions across different ecological regions. This review aims to systematically examine the global maize production landscape and abiotic stress-induced losses, analyze existing yield stabilization systems and their bottlenecks, and summarize the biological mechanisms by which AOSs regulate maize yield formation and stress tolerance. Furthermore, through an analysis of domestic and international research trends and bibliometric data, we identify future priority research directions for AOS-mediated enhancement of maize stress tolerance, providing theoretical foundations and practical pathways for the scientific application of AOSs in green and sustainable maize production.

## 2. Global Maize Production: Current Status, Threats, and Technical Bottlenecks

### 2.1. Current Status of Global Maize Production

Maize is the most widely cultivated and highest-yielding cereal crop globally, accounting for over 35% of global cereal production and serving as the staple food for approximately 1.2–1.5 billion people in sub-Saharan Africa, Latin America, and South Asia [16,17]. From 2005 to 2025, the global maize harvest area expanded from 146 to 207 million ha, production grew from 715 million to approximately 1.235 billion tons, and yield rose from 4.89 to about 5.97 t/ha (Figure 1). Sustaining this production is therefore central to global food security [18].

However, the growth in maize yield has clearly decelerated, with the average annual growth rate having fallen from about 2.5% in 1960–1990 to 1.5% in 1990–2010 and below 0.8% after 2010, with yield per unit area fluctuating around 5.85 ± 0.08 t/ha since 2016 [19,20]. Correlation analysis (Table 1) further shows that the correlation between yield and production declined from 0.9324 (2005–2015) to 0.5019 (2016–2025), indicating that recent production gains have relied increasingly on area expansion rather than yield improvement [21,22]. Traditional yield-enhancement pathways thus appear to be approaching diminishing returns, underscoring the need for new technologies to break this bottleneck.

### 2.2. Serious Threats of Abiotic Stress to Maize Production

Global warming, with a mean temperature rise of about 1.1 °C above pre-industrial levels and more frequent extreme weather, is increasingly threatening maize—which, despite its C4 photosynthetic efficiency, is highly sensitive to temperature and water availability during reproduction [23,24]. Among the major abiotic stresses, drought, heat, salinity, and waterlogging are the most damaging and frequently co-occur.

#### 2.2.1. Impact of Drought Stress

Drought is the primary abiotic stress limiting maize yield, with about 20–25% of the global maize area affected annually and conditions intensifying across roughly 72% of maize-growing regions [25,26]. Region-specific yield reductions range from 7.57% to 88.26%, with the largest declines occurring in Africa and South America (Table 2) [27,28,29,30,31,32,33,34,35,36,37,38]. Projections indicate that drought probabilities in major maize regions will remain high (37–84%) at mid-century, with yield losses approaching 50% in South America by 2050 [39,40].

#### 2.2.2. Impact of Heat Stress

Heat stress reduces maize yield by 32.7–40.9%, primarily by impairing pollen viability and silk fertilization once daily maxima exceed 35 °C during flowering, which sharply lowers kernel number and can account for up to 23% of total yield loss [41,42,43,44]. The frequency of flowering-stage heat damage in China’s Huang–Huai–Hai region has risen from once every 5–7 years (1980s) to once every 2–3 years (post-2010) [45,46]. Critically, heat is increasingly co-occurring with drought, and this compound stress now outweighs individual events as the dominant threat—it has triggered yield penalties of up to 30% in major maize regions since around 2000, and its frequency and intensity are projected to keep rising [47,48,49,50,51,52,53].

#### 2.2.3. Impact of Salinization Stress

Soil salinization and saline-water irrigation affect about 10 million ha of maize, imposing osmotic stress and ion toxicity that suppress emergence, vegetative growth, and kernel formation [54,55,56,57,58,59,60]. Maize is moderately salt-sensitive, with yield beginning to decline once soil electrical conductivity exceeds 1.7 dS/m and then falling by about 12% with each additional 1 dS/m, with combined water-and-salt stress reducing yield by 6.9–38.7% [60,61,62].

#### 2.2.4. Impact of Waterlogging Stress

Waterlogging, driven by extreme rainfall and poor drainage, damages maize throughout the growth cycle, with the jointing stage being most sensitive: submergence then prolongs the anthesis–silking interval and suppresses young-ear differentiation, reducing the kernel number by 6.7–44.5% and yield by 9.9–57.3% [63,64,65,66,67]. Regionally, monsoon rainfall makes China’s Huang–Huai–Hai region particularly vulnerable—the 2021 Henan flood alone cut summer-maize yield by 17–22% [62]. Overall, these four discussed stresses—drought, heat, salinization, and waterlogging—frequently co-occur and interact synergistically, so their combined impact far exceeds the sum of their individual effects (Table 3). Model-based estimates place the comprehensive annual yield loss at 250–350 million tons (20–30% of production), with an increasing trend under climate change [68,69,70].

### 2.3. Technical Approaches and Bottlenecks for Global Maize Yield Enhancement and Stabilization

Current strategies to counter these stresses fall into three broad pathways: genetic improvement, agronomic optimization, and chemical regulation, each of which faces distinct limitations.

#### 2.3.1. Genetic Improvement

Genetic improvement has driven most historical yield gains, but its progress in improving abiotic stress tolerance is lagging. Drought and salt tolerance are polygenic quantitative traits with small individual gene effects and strong genotype-by-environment interactions, so introgression of candidate genes, such as ZmNAC111 and ZmARGOS8, has delivered only modest (5–10%) and environment-dependent yield gains [10,76,77]. Long breeding cycles (8–10 years) and, for gene editing, unresolved issues such as multigene regulation, regulatory approval, and public acceptance further slow the response to rapidly changing climate stress [7,78,79].

#### 2.3.2. Agronomic Optimization

Agronomic optimization—including water-and-fertilizer integration, high-density planting, and conservation tillage—can substantially close the yield gap, but it is constrained by high cost and weak transferability. Drip fertigation, for instance, raises nitrogen-use efficiency and enables ultra-high yields, but its capital requirement (RMB 15,000–30,000/ha) is unaffordable for smallholders, and high-density planting aggravates lodging risk and late-season nutrient deficiency [80,81,82,83,84]. These technologies thus remain difficult to replicate across diverse, resource-limited cropping systems.

#### 2.3.3. Chemical Regulation and Biostimulants

Plant growth regulators such as ethephon and chlormequat can control plant architecture but have narrow application windows and carry the risk of suppressing growth; brassinosteroids and abscisic acid induce stress-responsive genes but are costly and short-lived in the field [12]. By contrast, biostimulants—defined as substances that stimulate plant nutrition and stress tolerance independently of nutrient content—are derived from natural raw materials, activate endogenous physiological processes, and are compatible with existing cultivation systems [85,86]. Their main limitation is a lack of clear molecular targets and rigorous dose–response validation, which causes variable field efficacy [87,88].

In summary, the core bottleneck is that these three strategic pathways have developed largely independently, with strong genotype–environment–management interactions affecting their performance and limited translation from laboratory mechanisms to field-operable solutions. A common direction for overcoming this bottleneck is the development of novel inputs that combine signal-regulatory function, low cost, broad adaptability, and environmental compatibility—requirements that alginate oligosaccharides (AOSs) are well suited to meet, as elaborated in the following sections.

## 3. Structure and Structure–Activity Relationships of Alginate Oligosaccharides

### 3.1. Sources of Alginate Oligosaccharides

AOSs are natural small-molecule compounds produced by the decomposition of brown algae in the ocean. These compounds act as signaling molecules that guide the stress-response behavior of algal communities. Currently, AOSs are primarily obtained through two approaches (Figure 2). One involves the degradation of natural polysaccharides extracted from brown algae (alginic acid, a linear copolymer composed of guluronic acid (G) and mannuronic acid (M) units). Degradation methods for obtaining AOSs from alginic acid include enzymatic hydrolysis, acid hydrolysis, and oxidative degradation [68]. Enzymatic hydrolysis is currently the most widely used process for preparing high-purity AOSs. Alginate lyase can specifically cleave the 1,4-glycosidic bonds in alginate molecules, producing oligosaccharide products with controllable degrees of polymerization under mild conditions (pH 6.5–8.0, temperature 30–50 °C). Common enzyme-producing microorganisms include marine bacteria such as *Flavobacterium*, *Pseudoalteromonas*, and *Vibrio* [89,90,91]. The degree of polymerization of enzymatic products is largely in the range of 2–8, which places them in the molecular weight interval with the strongest biological activity: when polymerization is too low (<2), signaling function is lost; when too high (>10), water solubility decreases and cell wall penetration becomes difficult [92]. Acid hydrolysis is less costly but involves harsh reaction conditions, poor selectivity, broad molecular weight distribution, and possible destruction of functional groups; consequently, it is gradually being replaced by enzymatic methods. Oxidative degradation uses oxidants such as H_2_O_2_ under alkaline conditions, generating products with more carboxyl and carbonyl groups and enhanced metal ion chelation capacity. These products can serve as functional fertilizer adjuvants, but their signaling activity in plants is typically weaker than that of enzymatic products.

Degradation yields oligosaccharides with a degree of polymerization (DP) of 2–8. These include homopolymeric guluronic acid structures, homopolymeric mannuronic acid structures, and heteropolymeric structures composed of both guluronic and mannuronic acids.

An alternative approach involves the direct degradation of brown algal powder via microbial fermentation to produce AOSs. Wang et al. (2024) used 5% (*w*/*v*) brown algal powder as the sole carbon source in fed-batch fermentation, obtaining a total AOS concentration of 28.5 g/L in the fermentation broth after 36 h, with the product DPs concentrated within 2–6 [93]. This powder-based method eliminates alginate extraction and purification steps, significantly simplifying the process flow. However, it currently faces bottlenecks, including obstruction of enzyme–substrate contact by non-alginate components in brown algal powder and mass transfer limitations due to the high viscosity of the fermentation broth.

### 3.2. Structural Features and Structure–Activity Relationships for Biological Activity

The biological activity of alginate oligosaccharides is closely related to their structural features, and structure–activity relationship studies are fundamental to understanding their functions (Figure 2). First, the DP is the primary determinant of activity. Studies using Arabidopsis and rice as model systems have shown that oligosaccharides with DPs of 4–8 have the strongest ability to promote root hair elongation and activate defense gene expression, whereas fragments with DPs <3 or >12 show significantly reduced activity [13,15]. This “optimal DP” phenomenon suggests that AOSs may be recognized by specific receptor proteins with selectivity for molecular length.

Second, the ratio and sequence arrangement of mannuronic acid-rich (M-rich) versus guluronic acid-rich (G-rich) regions significantly influence activity bias. M-oligosaccharides tend to be more effective in activating plant immunity and inducing antioxidant enzyme expression, which may relate to the greater flexibility of M blocks in forming linear conformations favorable for receptor binding. In contrast, G-oligosaccharides tend to have stronger metal ion chelation capacity, performing better in alleviating heavy metal stress [92,94]. Furthermore, unsaturated termini (enzymatic products often generate 4,5-unsaturated uronic acids at the non-reducing end) may also influence signal recognition efficiency, with some studies suggesting that unsaturated termini enhance AOS affinity for plant lectin-like proteins [92].

Third, chemical modification can expand functionality. Sulfated, acetylated, or metal-ion-chelated AOS derivatives exhibit additional value in bacteriostasis, water retention, and controlled release, providing opportunities for customized functional applications of AOSs [95]. However, field applications still predominantly use unmodified enzymatic products due to their optimal cost-effectiveness and verified safety. In summary, the biological activity of AOSs is not the effect of a single compound but an integrated manifestation of DP distribution, M/G composition, and terminal structure. Controlling these parameters during large-scale production is key to ensuring consistent field performance.

## 4. Possible Mechanism of Alginate Oligosaccharide Regulation of Maize Stress Tolerance

### 4.1. For Yield

Appropriate concentrations of AOSs can enhance maize grain yield through multiple pathways, as validated across different varieties, ecological regions, and application methods. Qu et al. (2020) investigated the mechanism of a seaweed biostimulant’s effects on yield formation in the maize variety “Denghai 605.” They found that the seaweed biostimulant increased the net photosynthetic rate and transpiration rate, with the 100-kernel weight increasing by approximately 5% [96]. Liu et al. (2025) applied 100 mg/L AOS compound fertilizer to the maize variety “Zhongdan 909” and found that, compared to compound fertilizer without AOSs, the 1000-kernel weight increased by 9.12%, stem diameter increased by 3.97–12.98%, and kernel number per ear increased by 2.21–8.32% [97]. Zhang et al. (2013) compared maize growth with different concentrations of alginate oligosaccharides and found that, compared to the control (peptide fertilizer), AOS-treated maize yield increased by 7.12% [98]. Pei et al. (2024) performed a meta-analysis of seaweed fertilizer application effects on crops (maize, wheat, rice) in China and found an average yield increase of 15.17% [99]. Guo et al. (2023) found that, after AOS application, maize grain yield increased by 16.2% and 15.7% across two growing seasons [100]. Notably, the “low-dose promotion, high-dose inhibition” characteristic of the dose–response curve is a pattern that must be carefully considered in AOS field application (Figure 3). At low concentrations, signal recognition and transduction efficiency increase with concentration; concentrations beyond the receptor saturation threshold may have negative effects, such as signal desensitization and excessive consumption of cellular reducing power [95,99,100,101]. Therefore, for AOS application to promote maize growth, precise control of the applied concentration (i.e., the concentration of the AOS solution) is significantly more important than controlling the total dosage.

In practice, AOSs are commonly delivered to maize through four application methods: (i) seed soaking or seed coating, in which seeds are immersed in or coated with an AOS solution (typically 50–500 mg/L) before sowing to promote germination and seedling vigor; (ii) foliar spraying, in which a dilute AOS solution (commonly 50–200 mg/L) is applied to the canopy at key growth stages (seedling, jointing, and early grain filling); (iii) root drenching or drip fertigation, in which AOSs are delivered directly to the root zone through irrigation systems at concentrations of 10–100 mg/L; and (iv) incorporation into compound or enhanced-efficiency fertilizers, in which AOSs are blended with basal or top-dressing fertilizers to achieve sustained release and synchronized nutrient uptake. The optimal method and concentration depend on the target stress, the growth stage, and the application equipment available.

### 4.2. For Drought Tolerance

AOSs enhance maize drought tolerance through synergistic actions across three levels: physiological protection, osmotic regulation, and morphological adaptation. The first level involves the activation of the antioxidant defense system. Under drought stress, excessive accumulation of reactive oxygen species (ROS) in plant cells leads to membrane lipid peroxidation and protein oxidative damage, with malondialdehyde (MDA) content serving as a key indicator of membrane damage [102,103]. Qu et al. (2026) demonstrated that seaweed biostimulants promote photosynthesis and respiration rates in maize leaves [95]. Furthermore, Guo et al. (2023) found that AOSs significantly increased maize leaf photosynthesis and shoot growth under drought conditions, resulting in a 15% increase in leaf area and a 17% increase in dry matter accumulation [100]. These findings indicate that AOSs maintain high antioxidant capacity throughout the maize life cycle, effectively protecting the photosynthetic apparatus from oxidative damage.

The second level involves the accumulation of osmotic regulatory substances. Proline and soluble sugars act as important osmoprotectants in the plant drought response by reducing cellular osmotic potential, maintaining turgor pressure, and functioning as molecular chaperones to protect protein structure [104]. Behboud et al. (2021) reported that 1% AOS treatment significantly increased proline and soluble protein content in maize seeds, thereby enhancing the germination rate and seedling growth [105]. Zhang et al. (2019) demonstrated that AOSs promote proline synthesis in Arabidopsis thaliana by upregulating P5CS1 gene expression; although this pathway has also been inferred in maize roots, direct molecular confirmation in maize is still limited, so this conclusion should be extrapolated with caution [15]. Additionally, leaf water potential in drench-treated plants was 0.35 MPa higher than in controls, indicating that AOS-treated plants possess greater water retention capacity [100].

The third level comprises adaptive changes in root morphology. AOS-promoted root growth and downward elongation provide an important structural basis for enhanced drought tolerance. Zhang et al. (2016) first confirmed in rice (*Oryza sativa*) that AOSs promote auxin synthesis by upregulating *OsYUCCA1* gene expression, thereby increasing lateral root density and root hair length [13]. This finding was obtained in rice, and, although a comparable auxin-mediated mechanism is plausible in maize, it has not yet been directly demonstrated in maize and should therefore be regarded as extrapolated evidence. Jindal et al. (2026) proposed that this adaptive response is primarily mediated through AOS-induced activation of abscisic acid (ABA) signaling pathways to improve drought recovery capacity [106]. AOS drenching increased root activity by 28.3% and enhanced water consumption in deep soil layers (40–60 cm), indicating improved root depth penetration and water uptake capacity [96]. This source expansion strategy, combined with stomatal conductance optimization (a conservation strategy that reduces ineffective transpiration), enables AOS-treated plants to maintain higher photosynthetic rates and water use efficiency under drought stress.

### 4.3. For Salt and Alkali Tolerance

Salt–alkali stress causes damage to maize through three key pathways; namely, osmotic stress (reducing rhizosphere water potential and inhibiting water uptake), ion toxicity (accumulation of Na^+^ and Cl^−^ in the cytoplasm, interfering with enzyme activities and metabolic processes), and oxidative stress (secondary stress) [105,106]. AOSs comprehensively alleviate this triple damage through core mechanisms involving the restoration of ion homeostasis and the maintenance of redox balance.

At the ion regulation level, AOSs promote Na^+^ efflux and restrict its long-distance transport to shoots by enhancing plasma membrane H^+^-ATPase activity and upregulating the expression of the Na^+^/H^+^ antiporter gene *SOS1*. Zhao et al. (2022), working with wheat (*Triticum aestivum*), found that AOS treatment significantly increased the expression levels of root TaSOS1, TaHKT1, and TaHKT5 under salt stress, reducing leaf Na^+^ content by 28% and increasing the K^+^/Na^+^ ratio by 45% [101]. These findings were obtained in wheat rather than maize; similar regulatory mechanisms have only been preliminarily validated in maize, and field trials showed that AOS drenching reduced maize leaf Na^+^ content by approximately 24% and increased K^+^ content by approximately 18.5%, significantly improving the K^+^/Na^+^ balance. Nevertheless, direct molecular confirmation of the SOS1/HKT pathway in maize remains incomplete, and the wheat-derived evidence should be interpreted as supporting rather than conclusive. Notably, the carboxyl and hydroxyl groups in AOSs possess metal ion chelation capacity, theoretically enabling the direct chelation of some Na^+^ in the rhizosphere to reduce root surface Na^+^ load. However, the contribution of this physicochemical effect is relatively limited under field conditions, with physiological regulation remaining dominant.

At the redox balance level, the magnitude of AOS-induced activation of the antioxidant system under salt stress is even greater than under drought stress. This suggests that the secondary oxidative burst triggered by ion toxicity is more severe than that induced by drought and indicates that AOS-induced antioxidant activation exhibits strong elastic responsiveness. The AsA-GSH cycle is a key pathway for plant H_2_O_2_ scavenging, and its operational efficiency determines cellular redox status. Related field trials (conducted in maize) showed that AOS drenching increased ear-leaf ascorbate peroxidase (APX) and glutathione reductase (GR) activities by approximately 25% and 30%, respectively, while the GSSG/GSH ratio decreased, indicating a shift toward a more reduced redox balance. This fine regulation enables cells to maintain the signaling function of H_2_O_2_ while avoiding its accumulation to toxic levels, representing an important feature that distinguishes the AOS salt tolerance mechanism from that of conventional antioxidants.

### 4.4. For Lodging Resistance

Maize lodging represents a serious challenge in high-density planting systems. The role of AOSs in enhancing stalk strength is a recent discovery that provides new insights for addressing this traditional problem. Field trials by Chen et al. (2024) showed that AOS drenching at the elongation stage increased the puncture strength of the third basal internode by 21.5% and reduced the stalk breakage rate by approximately 30%, with no significant changes in plant or ear height. This indicates that AOS-induced stalk strengthening is not achieved through the inhibition of vegetative growth but through the direct improvement of the stalk’s mechanical tissue construction [107].

Lignin is a key component conferring mechanical strength to stalks, and its synthesis is precisely regulated by the phenylpropanoid metabolic pathway. Phenylalanine ammonia-lyase (PAL) is the first rate-limiting enzyme in the lignin synthesis pathway, while 4-coumarate-CoA ligase (4CL) and cinnamyl alcohol dehydrogenase (CAD) catalyze midstream and downstream steps, respectively. Through transcriptome analysis, Li et al. (2022) found that within 24 h of AOS drenching, the expression levels of the *ZmPAL*, *Zm4CL*, and *ZmCAD* genes in maize stalks were upregulated 2.8-, 2.1-, and 3.3-fold, respectively [96]. Correspondingly, lignin content in the third basal internode increased by 13.6%, cellulose content increased by 9.8%, and vascular bundle sheath cell wall thickness increased significantly, as observed microscopically. This effect may relate to AOS regulation of the dynamic balance between auxin and gibberellin. Moderate auxin signaling promotes vascular bundle differentiation and secondary wall deposition, while fine-tuning of gibberellin levels helps maintain the appropriate ratio of mechanical to parenchymal tissues in internodes [108].

The stalk-strengthening effect of AOSs not only reduces the lodging rate but also indirectly benefits yield formation and mechanized harvesting efficiency. Lodged plants suffer from disrupted photosynthetic canopy structure, interrupted grain filling, and increased susceptibility to ear mold due to their proximity to the ground. Reducing lodging avoids these indirect losses; in high-density, high-yield fields, the contribution of lodging resistance to yield preservation may be comparable to the direct yield-increasing effect.

### 4.5. Synergistic Mechanisms for Multiple Stress

The benefits of AOSs for maize extend beyond enhanced stress tolerance, demonstrating diverse growth-promoting effects. Figure 4 presents a diagram of the multi-mechanism synergistic network of AOS action on maize. Regarding emergence and early seedling vigor, AOS seed soaking improves the germination potential and germination index under low-temperature stress, which is related to the promotion of α-amylase activity and accelerated mobilization of storage materials in seeds [100]. Regarding nutrient use efficiency, Zhang et al. (2017) found that alginate oligosaccharides increased chlorophyll content and net photosynthetic rate in functional maize leaves while also promoting nitrogen, phosphorus, and potassium uptake. Under the same nitrogen application conditions, the application effects were superior to those of other enhanced-efficiency nitrogen fertilizers, increasing nitrogen fertilizer recovery by approximately 20% [109]. The root-level mechanism is more direct: AOSs can upregulate the expression of the nitrate transporter genes *ZmNRT1.1B* and *ZmNRT2.2* in maize roots, thereby enhancing roots’ capacity to capture soil nitrate nitrogen.

In conventional maize, increased 100-kernel weight and improved grain test weight indirectly reflect enhanced grain filling. Additionally, multiple trials have shown that AOSs delay ear-leaf senescence, manifested as maintained chlorophyll content and photosynthetic rate during late grain filling. This provides the material basis for extending the effective grain-filling period and increasing grain weight [96].

Regarding heavy metal stress, Vivodová et al. (2023) used transcriptomic analysis to elucidate the mechanism by which AOSs alleviate cadmium toxicity in maize. They found that AOSs upregulate metallothionein genes and glutathione synthesis-related genes, thereby enhancing cadmium compartmentalization and detoxification [110]. Furthermore, AOS carboxyl groups promote chelation and precipitation of cadmium ions. This dual action suggests that AOSs have potential application value for safe production on heavy-metal-contaminated farmland.

## 5. Bibliometric Analysis and Domestic and International Research Trends for Alginate Oligosaccharides

### 5.1. Literature Association Networks and Research Hotspot Evolution

To quantitatively characterize the knowledge structure and dynamic evolution of the AOS–maize research field, we searched the Web of Science and CNKI databases for the period 2005–2025 using the search terms “alginate oligosaccharide” OR “AOS” AND “maize” OR “corn” (and their Chinese equivalents). After deduplication, only 10 valid documents were retrieved, indicating that AOS–maize research remains in a preliminary stage. According to these articles, all authored by Chinese scholars, domestic research currently holds a leading position globally in the field of AOS-promoted maize growth. Overall, current research on AOS–maize remains limited, with insufficient accumulated data. It should be emphasized that, owing to the very small sample size (10 documents), the bibliometric maps generated by VOSviewer (version 1.6.21) in this study are inherently limited in their statistical robustness and generalizability. The clustering and time-zone patterns described below should therefore be interpreted as a preliminary depiction of an emerging field rather than as a definitive or exhaustive characterization and should be placed within the broader context of AOS research in model plants and other crops.

Although the co-occurrence network in Figure 5 displays multiple clusters, they can be broadly categorized into three closely related research subgroups. Cluster 1: Fertilizer synergism and nutrient management. This is the most prominent and well-supported cluster in the dataset, with core keywords including “alginate oligosaccharide,” “enhanced-efficiency nitrogen fertilizer,” “fertilizer use efficiency,” “NPK use efficiency,” and “compound fertilizer.” Studies indicate that adding alginate oligosaccharides to fertilizers (e.g., alginate oligosaccharide urea, AOS-containing N–P–K compound fertilizer) significantly improves nitrogen, phosphorus, and potassium uptake efficiency in maize and other crops, thereby reducing fertilizer while maintaining or increasing yield. Cluster 2: Crop physiological regulation and yield/quality enhancement. This cluster focuses on the effects of alginate oligosaccharides on crop physiology, with keywords including “maize,” “seedling growth,” “photosynthetic characteristics,” “chlorophyll fluorescence,” and “yield.” The literature confirms that alginate oligosaccharides increase chlorophyll content and net photosynthetic rate (Pn) in functional leaves, promoting above- and belowground biomass accumulation and ultimately increasing kernel number per ear, 1000-kernel weight, and yield. Cluster 3: Comprehensive applications and mechanistic reviews. This cluster comprises multiple review articles, with keywords including “bioactive substances,” “stress resistance,” “plant growth regulation,” and “green agriculture.” These articles systematically review the multifunctional roles of alginate oligosaccharides, including enhanced stress tolerance (drought, cold, and salt tolerance), induced disease resistance, and regulation of plant growth and development as biostimulants, emphasizing their strategic value in promoting green agricultural development.

The research trajectory in this field can be divided into three phases. Phase 1 (2013–2017): Empirical exploration of fertilizer synergism. During this phase, “alginate oligosaccharide-enhanced fertilizer (NPK)” and “enhanced-efficiency nitrogen fertilizer” were core burst keywords. Research in this phase focused on quantitatively validating, through pot and field trials, the direct growth-promoting effects of alginate oligosaccharides as fertilizer additives on maize, with comparisons to conventional synergists (e.g., peptides). Phase 2 (2016–2021): Mechanistic inquiry and target expansion. Physiological indicators such as “photosynthetic characteristics” and “nutrient uptake” became research hotspots, with studies shifting from determining “whether it works” to elucidating “how it works.” Application methods also diversified, with new treatments such as “seed coating” emerging. Phase 3 (2022–present): Systematic summarization and green orientation. The concentrated appearance of multiple review articles marks the field’s entry into a stage of systematic summarization and strategic outlook. “Green agriculture” and “yield increase with quality improvement” became high-frequency burst keywords, reflecting the close alignment of research orientation with national agricultural policies focused on fertilizer reduction, efficiency enhancement, and green development.

### 5.2. Research Gaps and Bottlenecks

In terms of research volume and structure, China accounts for approximately 85% or more of global publications on AOS–maize application research. This distribution pattern is highly consistent with China’s brown algae resource endowment, progress in the industrialization of enzymatic preparation, and maize production scale. Currently, research on alginate oligosaccharide–maize applications in China exhibits a distinctive “application-first” character, with strengths including adequate field validation, active product development, and broad ecological region coverage. However, there remains room for improvement in mechanistic depth and international collaboration, alongside multiple scientific and technical challenges.

(1) Lag in signal receptor identification. Although substantial evidence indicates that AOSs can activate plant pattern-triggered immunity (PTI) signaling, the specific receptor in maize has not yet been identified. Compared with the resolution of the chitosan oligosaccharide receptor AtCERK1, the lack of AOS-receptor identification seriously constrains an in-depth understanding of signal perception and early transduction mechanisms and precludes receptor-structure-based molecular design.
(2) Limited research on combined-stress mechanisms. Existing studies predominantly focus on AOS regulation of single stresses (drought or salinity), whereas combined stress is the norm in field production. Under conditions such as drought with high temperature or salinity with waterlogging, the regulatory priorities, pathway interactions, and possible antagonistic effects of AOSs remain unclear, limiting the optimization of application strategies in real field environments with complex conditions.
(3) Insufficient multi-year, multi-site networked data. Current field trials mostly rely on 1–2-year single-site data, making it difficult to evaluate treatment and year and location interaction effects. As AOSs are signaling molecules, their effects may be significantly modulated by environmental factors such as soil type, organic matter content, and rainfall distribution. The lack of long-term positioned trials and cross-ecological-region network data leads to questionable regional universality of recommended protocols.
(4) Low compatibility of formulation and application technology. Current AOS field application mostly uses conventional sprayers or simple injectors, with insufficient developments compatible with modern agricultural equipment, such as drip irrigation systems, simultaneous seed-and-fertilizer drilling machines, and unmanned aerial vehicle (UAV) spraying. The lack of specialized high-concentration stable liquid formulations, sustained-release microcapsules, and other formulations makes it difficult to integrate AOSs into the technical systems of large-scale farming operations. Addressing this gap requires the development of practical application protocols, including standardized formulations (e.g., high-concentration liquid concentrates and sustained-release granules), clear dose recommendations for each delivery method, and compatibility testing with existing agronomic systems, such as drip fertigation, seed treatment, and UAV-based spraying. Establishing such protocols is essential for translating laboratory findings into field-operable solutions compatible with modern, large-scale maize production.

### 5.3. Future Research Priorities

In response to the above challenges, and synthesizing research directions for AOSs in other maize systems along with the current domestic and international research status, we propose the following priority directions for breakthroughs in AOS–maize research. Figure 6 presents a roadmap for future research directions in AOS–maize research.

(1) Molecular decoding of AOS signal perception and transduction. CRISPR/Cas9 genome editing should be employed for the functional validation of candidate receptor-like kinase genes to identify the maize AOS receptor, with a particular focus on membrane protein families containing LysM and Malectin domains. Simultaneously, phosphoproteomics and single-cell transcriptomics should be used to map the comprehensive signaling response landscape of root tip cells following AOS treatment, thereby elucidating the complete cascade network from plasma membrane perception to nuclear transcriptional reprogramming. Resolving these mechanisms is expected to provide potential targets for the functional optimization and mimetic design of AOSs.
(2) Multi-stress synergistic regulation and application strategy optimization. Multi-year field trials spanning five or more consecutive years across diverse ecological regions should be conducted to evaluate AOS regulation under typical combined-stress conditions, such as “drought and heat” and “salinity and waterlogging.” Metabolomics and ionomics should be utilized to identify universal and stress-specific response biomarkers, systematically evaluate treatment-by-environment interaction effects and the carry-over effects of sustained application, and establish AOS application decision models based on stress type. For instance, it remains to be determined whether AOSs preferentially protect the photosynthetic system under combined drought–heat stress or focus on maintaining root ion barrier function under salinity–waterlogging conditions.
(3) Elucidation of rhizosphere microecological regulation mechanisms. Although AOS drenching inevitably affects rhizosphere microbial communities, this indirect growth-promoting pathway has been largely neglected. Combined metagenomic and metabolomic analyses should be employed to investigate whether AOSs selectively enrich beneficial bacteria, such as *Pseudomonas* and *Bacillus*; activate soil urease and phosphatase activities; and improve rhizosphere nutrient transformation. These studies will provide feasible pathways for revealing the mechanisms by which AOSs regulate the rhizosphere microbiome.

## 6. Conclusions

AOSs, a class of structurally defined marine-derived biostimulants with diverse activities, demonstrate significant potential and cross-ecological stability in promoting maize yield, enhancing drought and salt tolerance, and improving stalk lodging resistance. Their core mechanisms operate at multiple levels—signaling (activation of plant immune responses and auxin pathways), physiology (systematic enhancement of antioxidant defense and osmotic regulation), and morphology (optimization of root architecture and stalk mechanical tissue). Compared with other biostimulants, AOSs offer comprehensive advantages, including low effective concentrations, well-defined mechanisms, renewable raw materials, and compatibility with modern agricultural equipment. However, current AOS–maize research requires further advancement in terms of molecular mechanistic depth, combined-stress regulation, and long-term field validation. To address the global strategic demand for green enhancement of maize yield, future efforts should focus on resolving signaling receptors, elucidating multi-stress synergistic mechanisms, and regulating rhizosphere ecology. These initiatives will provide the data and mechanistic support necessary to bridge the gap from “scientific discovery” to “field solutions,” facilitating the integration of AOSs into technical systems of maize production to enhance stress tolerance and stabilize yields, thereby offering a sustainable pathway for global maize production.

## Figures and Tables

**Figure 1:**
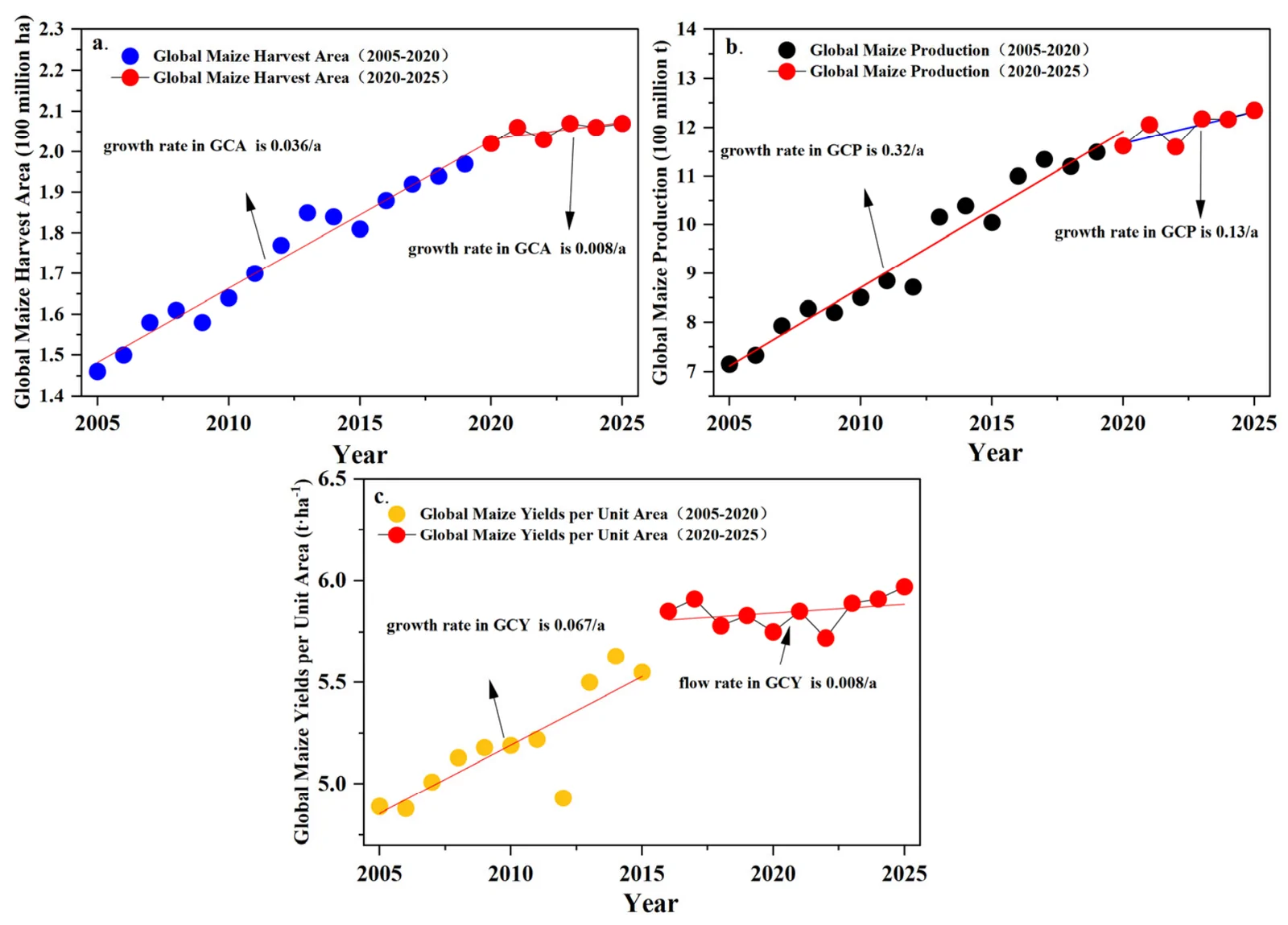
Trends in global maize harvest area, total production, and yield per unit area from 2005 to 2025. (**a**) Global maize harvest area (million ha) by year; (**b**) Global maize total production (million tons) by year; (**c**) Global maize yield (t/ha) by year. Data are derived from FAO statistical databases for 2005–2023, with 2024 and 2025 data based on USDA WASDE report projections. The sharp decline in 2012 was attributed to severe drought in the United States, while the decline in total production in 2022 was influenced by the Ukraine crisis and high temperatures in multiple regions.

**Figure 2:**
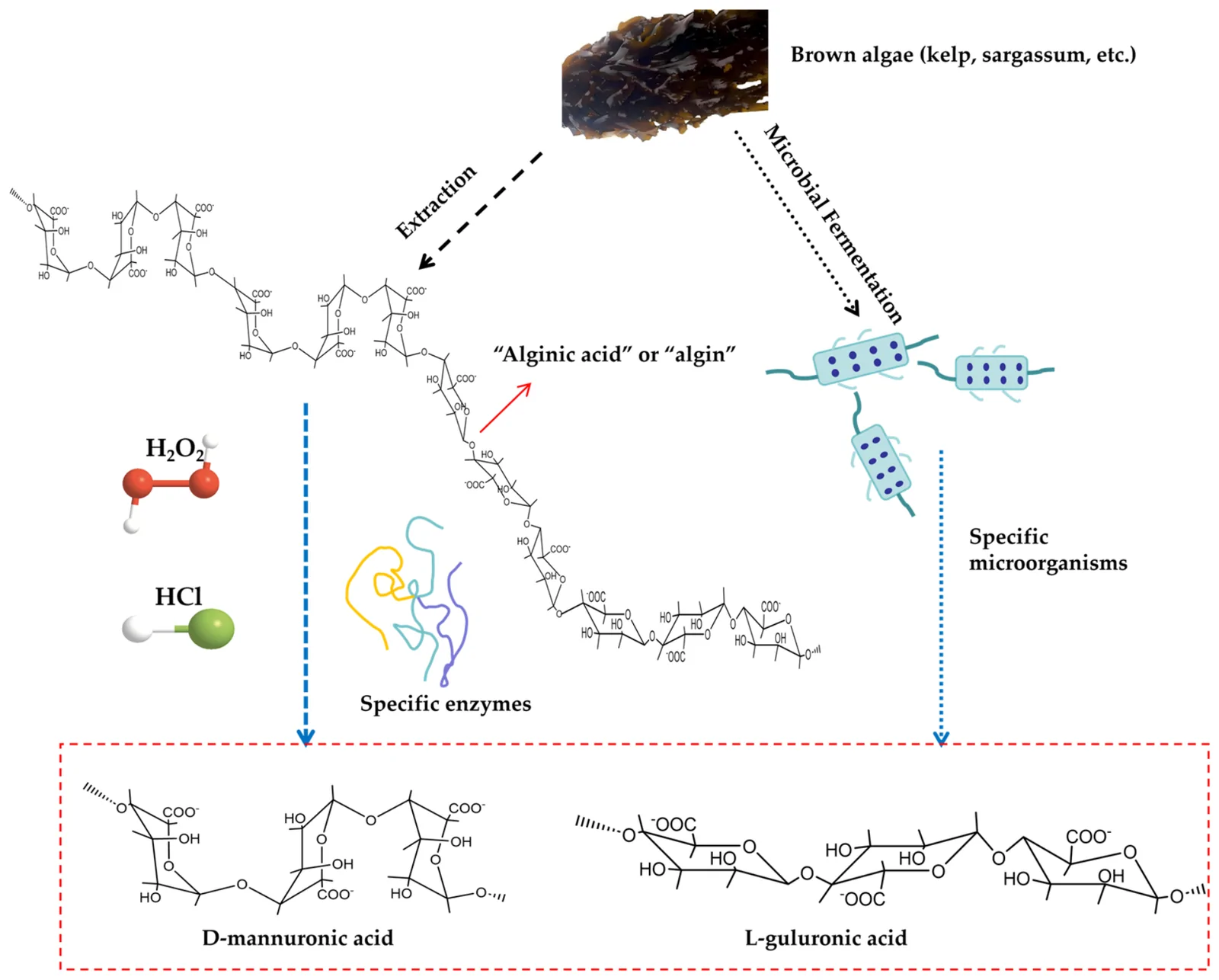
Alginate oligosaccharide preparation process and M/G structural features.

**Figure 3:**
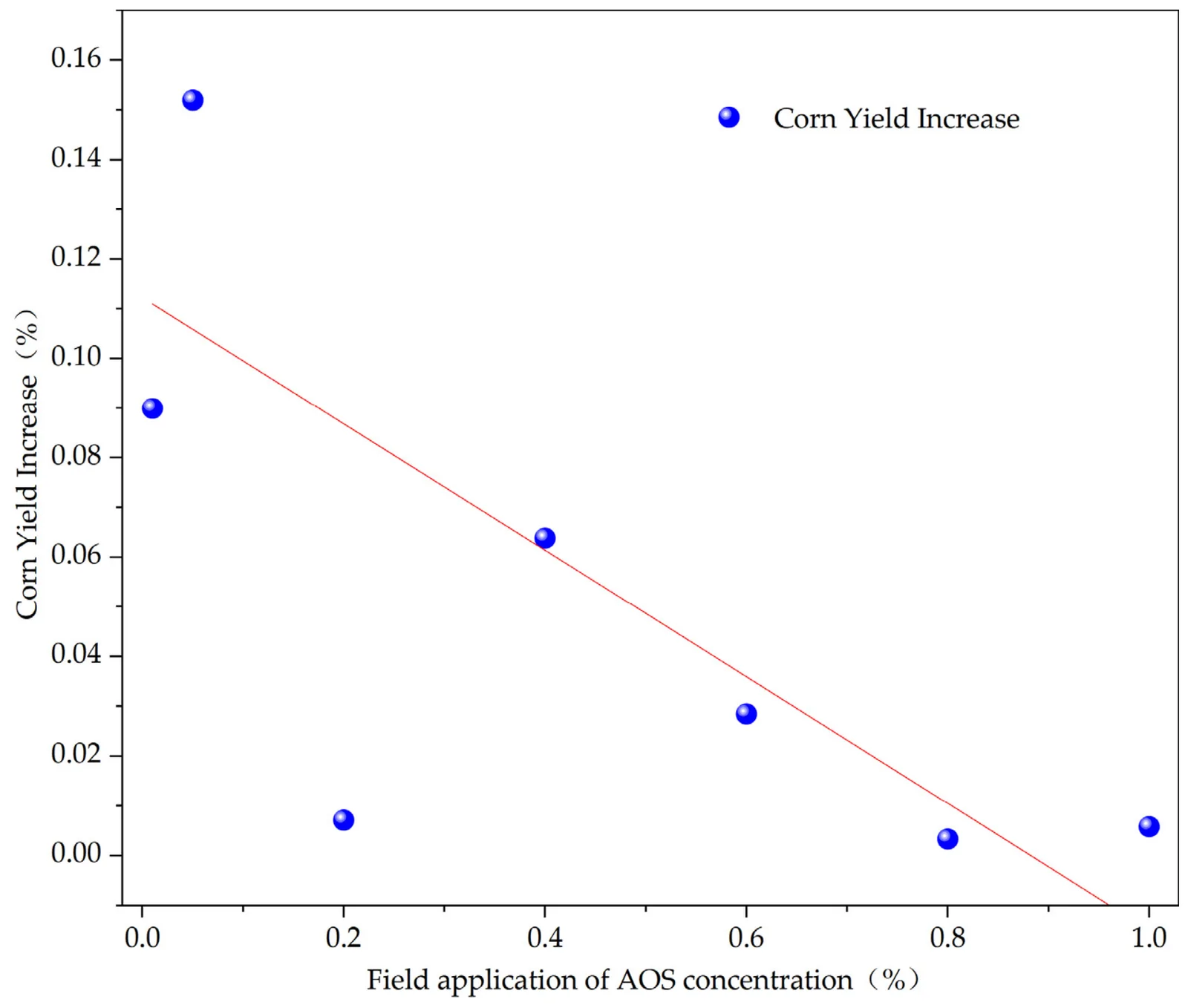
Line graph illustrating the relationship between AOS application concentration and maize yield increase, based on literature data [95,96,97,98,99,100].

**Figure 4:**
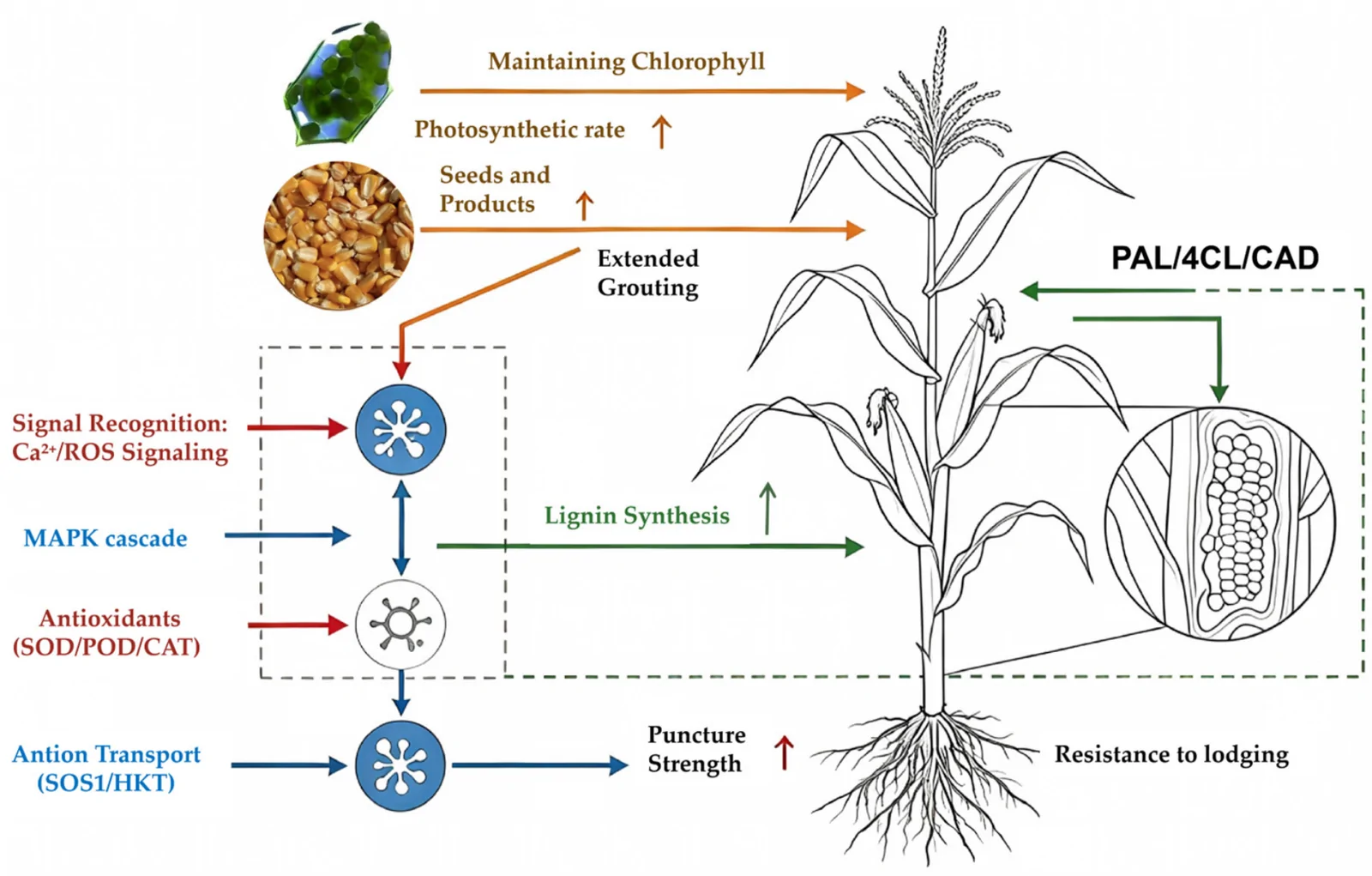
Multi-mechanism synergistic network diagram illustrating AOS regulation of maize stress tolerance and yield enhancement. Root-level mechanisms: signal recognition (receptor) → Ca^2+^/ROS signaling → MAPK cascade → antioxidant enzymes (SOD, POD, CAT) → ion transport (SOS1, HKT). Stalk-level mechanisms: PAL/4CL/CAD → increased lignin synthesis → increased puncture strength → lodging resistance. Leaf-level mechanisms: chlorophyll maintenance → increased photosynthetic rate → extended grain filling period. Red arrows indicate signal perception and transduction pathways; blue arrows indicate antioxidant and ion regulation pathways; green arrows indicate stalk strengthening pathways; orange arrows indicate carbon–nitrogen metabolism and quality improvement pathways.

**Figure 5:**
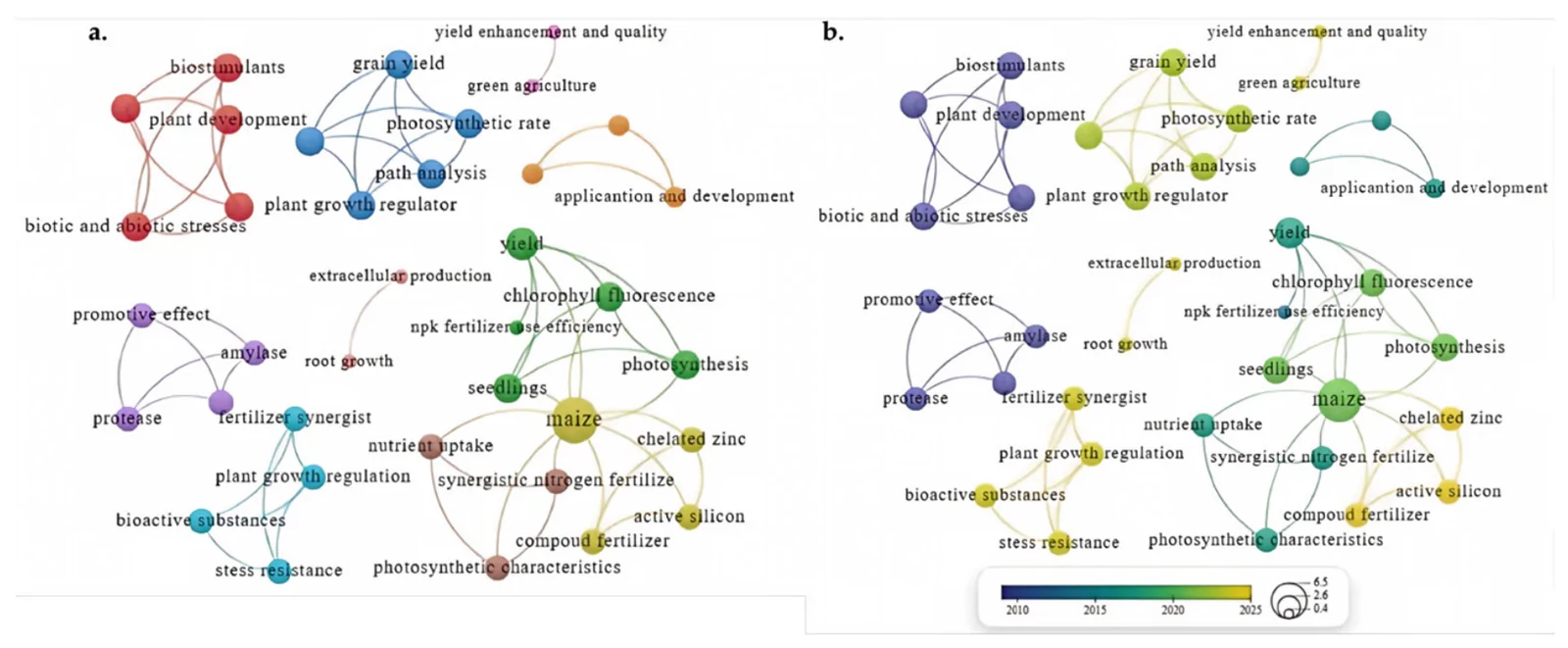
Bibliometric analysis of alginate oligosaccharide–maize research (CNKI and Web of Science). (**a**) Keyword co-occurrence cluster network; (**b**) Time-zone evolution diagram and key node burst detection.

**Figure 6:**
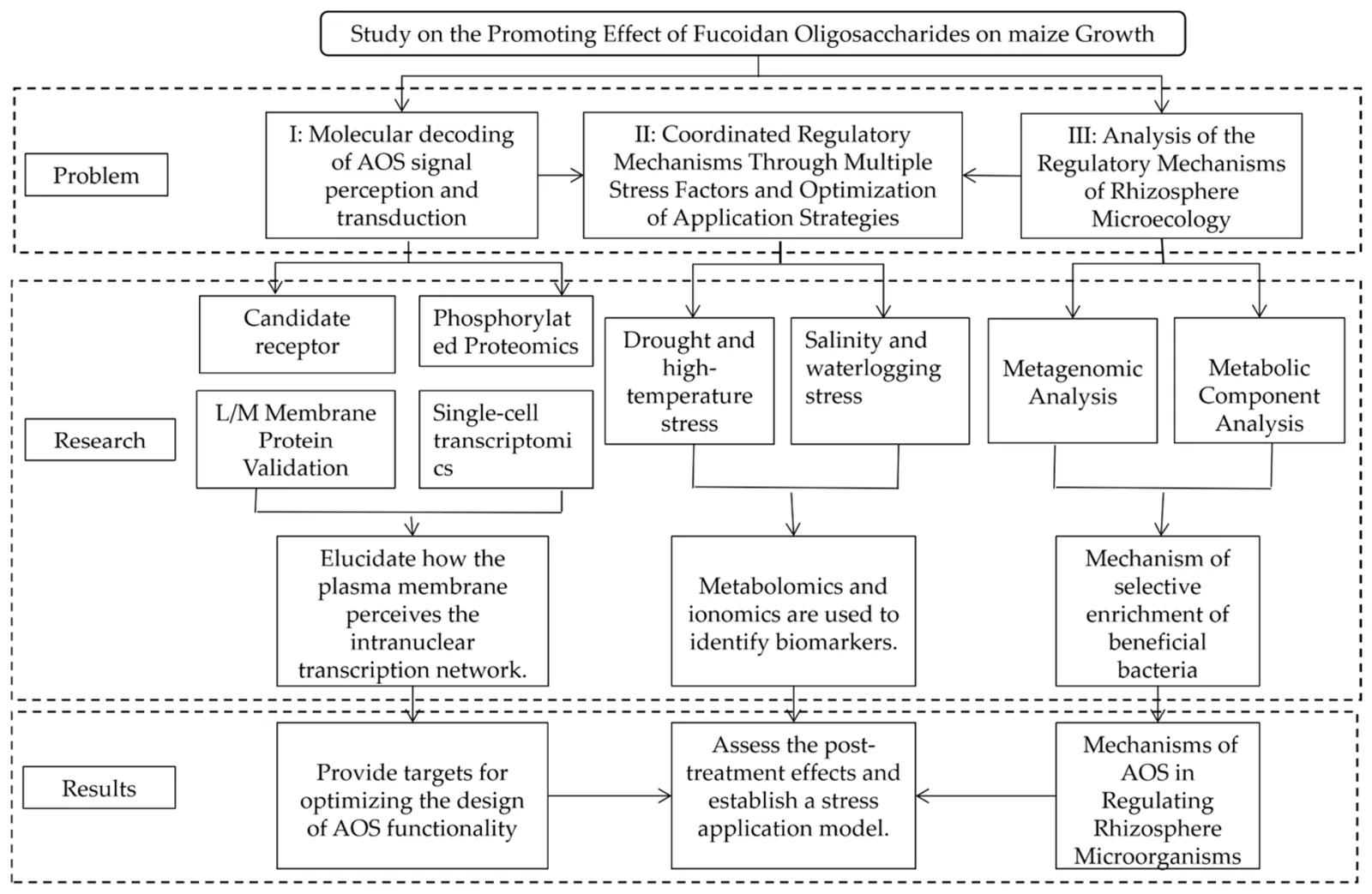
Future research roadmap for AOS–maize research.

**Table 1:** Correlation analysis among global maize yield, harvest area, and total production.

2005–2015	GCP	GCY	GCA
GCP	1	0.9324	0.9647
GCY	0.9323	1	0.8058
GCA	0.9646	0.8058	1
2005–2015	GCP	GCY	GCA
GCP	1	0.5019	0.9420
GCY	0.5019	1	0.1831
GCA	0.9420	0.1831	1

Note: Data used for correlation calculations are derived from FAO statistical databases for 2005–2023; 2024 and 2025 data are USDA WASDE report projections. GCP, global maize production; GCA, global maize harvest area; GCY, global maize yield. The second period label in the original table read “2005–2015” but has been corrected to “2016–2025” to match the description in the text.

**Table 2:** Effect of drought on maize production in major maize-producing regions over the past 20 Years.

Regions	The Reduction in Maize Production (%)	References
African	25–88.62%	[27,28]
South American	14–50%	[29]
China	14.8–35%	[30,32]
USA	7.57–42.7%	[33,34,35,36,37,38]

**Table 3:** Estimated average annual yield losses from abiotic stresses in global maize production, 2005–2024.

Stress Type	Average Annual Loss (Million Tons)	% of Total Production	Representative Events and Losses	References
Drought	1.2–1.8	10–15%	Midwest severe drought: yield dropped 25%, loss ~110 million tons, economic loss > $30 billion; 2020–2023 Horn of Africa: five consecutive failed rainy seasons, >20 million people facing food crisis, maize prices tripled	[7,71,72]
High Temperature	0.6–0.9	5–8%	2022 South Asia (India, Pakistan) extreme heat wave: flowering temperatures > 40 °C for multiple days, 20–30% yield reduction; Huang-Huai-Hai summer maize heat damage frequency increased from once per 5–7 years to once per 2–3 years	[25,45]
Salinity	0.4–0.6	3–5%	Nile Delta secondary salinization: 30–50% yield reduction; Yellow River Delta and Xinjiang salt-affected maize: 30–50% yield reduction due to poor emergence and premature senescence, complete failure in severe cases	[54,55,56,57,58,59,60]
Waterlogging	0.4–0.7	3–6%	2021 Henan extreme rainfall: >1 million ha affected, ~200,000 ha complete failure, loss ~15 million tons, direct economic loss > RMB 20 billion; 3–5 days flooding causes 20–30% reduction, >7 days causes complete failure	[73,74,75]
Total loss	2.5–3.5	20–30%	The above four stress types often occur as combined stresses (drought and heat, salinity and waterlogging), with synergistic damage far exceeding the sum of individual stresses; simple additive approaches overestimate	[9,68,69]

Note: Drought loss data were synthesized from global gridded crop model integrated analyses and USDA disaster assessment reports; high-temperature loss data were derived from global temperature–yield response functions and multi-country yield statistics; salinity loss data were estimated based on global salt-affected land distribution and average maize yield reduction rates; waterlogging loss data were integrated from EM-DAT records and field flooding simulation experiments. Total losses account for the non-additive nature of combined stress effects and are presented as comprehensive ranges.

## Data Availability

No new data were created or analyzed in this study. Data sharing is not applicable to this article.
